# Pro‐tumorigenic role of ERα in prostate cancer cells

**DOI:** 10.18632/aging.100769

**Published:** 2015-06-25

**Authors:** Luc Furic, Mitchell G. Lawrence, Gail P. Risbridger

The importance of estrogen actions in the etiology of prostate cancer (PC) has been widely accepted following the demonstration that androgens, in combination with estrogens, can induce neoplastic lesions in the prostate [1]. The tumor microenvironment is crucial in this process since tissue recombination assays showed that stromal ERα is required for neoplastic transformation. The apparent lack of epithelial ERα expression in the majority of PC previously led to the notion that direct estrogen action in tumor cells is only through ERβ, a paralog of ERα with antitumor activities. However, we recently reported ERα positive epithelial cells in approximately half of the patients with aggressive, high Gleason score tumors [2].

As we reported, not all epithelial cells in a given tumor are positive for ERα expression. Similar findings of heterogeneous ERα expression have been published recently in a distinct cohort of PC patients in which epithelial ERα expression correlates with poor patient prognosis [3]. What causes a subset of PC cells to express ERα is currently unknown. Previous work demonstrated that ERα expression can be silenced by promoter methylation, and it is likely that re-expression of ERα in PC cells involves chromatin modifications. With advances in cell sorting technology (using intracellular fluorescent probes) and progress in isolating viable cell populations directly from human prostate tissues, we are confident that gene expression analysis of ERα-positive PC cells is achievable and represents an important step towards understanding the pro-tumorigenic role of epithelial ERα in PC.

Alternatively, we generated an ERα specific gene expression signature from murine PTEN-null prostate epithelial cells and gene ontology analysis revealed that ERα regulates the expression of a wide range of mRNAs coding for proteins involved in carbohydrates metabolism [2]. The loss of PTEN facilitates downstream activation of mTORC1 and both events are associated with increased glycolytic and proliferative activity in tumors [4]. Our demonstration that ERα sustains this glycolytic environment [2] could be specifically associated with PTEN-loss and it will be important to assess the role of ERα in prostate tumors harboring different genetic aberrations (e.g TMPRSS2-ERG fusion, MYC overexpression, etc). In the context of the loss of PTEN, we also showed that downregulation of ERα decreases the proliferative potential of PC cells. This reduction in proliferation was accompanied by inhibition of the phosphorylation of ERK1/2, AKT and MNK, key kinases of the MAPK and PI3K pathways. This is in agreement with the “non-genomic” model of ERα action in breast cancer where a pool of ERα is associated with the plasma membrane to direct the activation of the MAPK and PI3K pathways. In addition, the ability of ERα to regulate gene expression at the transcriptional level, and to modulate the activity of signaling pathways that impinge on mRNA translation efficiency, potentially adds a layer of complexity to the gene expression program of ERα in cancer cells. This hypothesis was explored in breast cancer MCF7 cells and revealed that ERα directly regulates the translational efficiency of target mRNAs [5].

Recent results from Rubin & colleagues [6] not only support our findings that subsets of PC cells express ERα, but show that ERα regulates the expression of a long non coding RNA (NEAT1) which is associated with poor prognosis. ERα has also been shown to regulate the expression of multiple miRNAs in breast cancer cells [7]. Considering that our gene expression profiling was performed using microarrays representing only mRNAs, this precluded us from identifying the “noncoding” transcriptional profile of ERα. Future work to assess if miRNAs are regulated by ERα in PC will bridge the gap between transcriptional and translational profiling of PC cells expressing ERα.

Overall, these findings provide a framework in which further experiments could be designed to validate ERα as a biomarker of poor prognosis in PC or as a direct therapeutic target. This could lead to the preclinical testing of pure antagonists of ERα in ERα-positive PC.
